# Risk of children being taken into care amongst Metis parents experiencing incarceration: A linked administrative data study.

**DOI:** 10.23889/ijpds.v7i3.1945

**Published:** 2022-08-25

**Authors:** Emily Brownell, Julianne Sanguinsll, Jennifer Enns, Marni Brownell, Randy Walld, Farzana Quddus, Shannon Allard-Chartrand, Anita Durksen, Janelle Boram Lee, Lorna Turnbull, Marcelo Urquia, Alyson Mahar, Elizabeth Wall-Wieler, Hygiea Casiano, Nathan Nickel

**Affiliations:** 1 Manitoba Centre for Healthy Policy; 2 Manitoba Metis Federation; 3 University of Manitoba

## Objectives

Indigenous children in Canada, including Metis children, are taken into care by the child welfare system at a disproportionately high rate. The objective of this study was to explore factors related to having a child taken into care from Metis families in Manitoba, and specifically from Metis mothers experiencing incarceration.

## Approach

The cohort comprised all mother-child dyads in Manitoba, Canada with live births between Jan 1, 2004-Dec 31, 2014. The factors we examined included the mother experiencing incarceration after the birth of a child, having prior interaction with the legal system, socioeconomic status, maternal age, and mother’s mental health disorder diagnoses. Using the Cox proportional hazards model, unadjusted and adjusted hazard ratios describing the risk of a child being taken into care by five years of age were generated for three exposure groups: no incarceration, prenatal incarceration, and postnatal incarceration.

## Results

Before adjustment, mothers experiencing incarceration after the birth of a child had greater risk of having a child taken into care (HR 9.3; 95% CI 7.7-11.3) compared with women who did not experience incarceration; after adjusting for measured confounders, their elevated risk dropped to aHR 6.1 (5.0-7.4). After further adjustment for prior custody in the prenatal period, mothers experiencing incarceration had 3.8 times the risk (3.0-5.0), and after adjusting for maternal mental health disorders, this dropped to 2.6 times the risk (2.0-3.4). In fully adjusted models, other factors associated with having a child taken into care included receiving diagnoses for a substance use disorder (aHR 2.6; 2.1-3.2) and for psychosis (aHR 2.4; 1.4-4.1) during the five years prior to the child’s birth.

## Conclusion

Due to colonialism, Metis women experience high levels of social and mental health complexity, placing their children at increased risk of being taken into care. Responding to the Truth and Reconciliation Commission’s Calls to Action to redress the harms of colonialism is necessary to support Metis children’s health and well-being.

